# Detection of *Anaplasma* spp. and *Ehrlichia* spp. in dogs from a veterinary teaching hospital in Italy: a retrospective study 2012–2020

**DOI:** 10.1007/s11259-024-10358-4

**Published:** 2024-03-27

**Authors:** Veronica Facile, Maria Chiara Sabetti, Andrea Balboni, Lorenza Urbani, Alessandro Tirolo, Martina Magliocca, Francesco Lunetta, Francesco Dondi, Mara Battilani

**Affiliations:** 1 Department of Veterinary Medical Sciences, Alma Mater Studiorum-University of Bologna, Via Tolara di Sopra 50, Ozzano dell’Emilia, Bologna, 40064 Italy; 2 Department of Veterinary Sciences, University of Parma, Strada del Taglio 10, Parma, 43126 Italy

**Keywords:** Diagnosis, PCR, Phylogeny, Serological tests, Tick-borne pathogen

## Abstract

*Anaplasma phagocytophilum*, *Anaplasma platys* and *Ehrlichia canis*, responsible of diseases in dogs, are tick-borne pathogens with a proven or potential zoonotic role that have shown increasing prevalence worldwide. The aims of this retrospective study were to assess the frequency of *Anaplasma* spp. and *Ehrlichia* spp. exposure in dogs tested in a veterinary teaching hospital in Italy over a 9-year period, to compare the performance of the diagnostic tests used, to evaluate correlations with clinical data, and to genetically analyse the identified bacteria. During the study period, 1322 dogs tested by at least one of the rapid immunoenzymatic test, indirect immunofluorescent antibody test or end-point PCR assay for *Anaplasmataceae* detection were included. Dogs were tested if they had clinical signs or clinicopathological alteration or risk factors related to infection, and if they were potential blood-donor animals. Ninety-four of 1322 (7.1%) dogs tested positive for at least one pathogen: 53 (4.3%) for *A. phagocytophilum*, one (0.1%) for *A. platys* and 63 (4.6%) for *E. canis*. The number of dogs tested increased and the positivity rate progressively declined over the years. Comparison of tests showed a near-perfect agreement between serological tests and a poor agreement between PCR and indirect assays. A breed predisposition has been highlighted for *A. phagocytophilum* infection in hunting breed dogs and for *E. canis* infection in mixed breed dogs. Phylogeny confirmed potential zoonotic implications for *A. phagocytophilum* and showed no correlation of the identified bacteria with the geographical origin. Our study provides new insights into possible risk factors in dogs and evidenced discordant results between different tests, suggesting that a combination of serological and molecular assays is preferable for a correct diagnosis.

**Supplementary Information:**

The online version contains supplementary material available at 10.1007/s11259-024-10358-4.

## Introduction

In a scenario of increased density and geographical expansion of ticks, due to climate change and global warming, and of increased animal traveling and more frequent contact between wild and domestic species, tick-borne pathogens (TBP) are gaining importance due to their huge impact on animal and human health (Sanchez-Vicente et al. 2019; Wilson et al. 2020).

*Anaplasma phagocytophilum, Anaplasma platys* and *Ehrlichia canis*, which are responsible for granulocytic anaplasmosis, canine infectious cyclic thrombocytopenia and canine monocytic ehrlichiosis (CME), respectively, are three examples of TBP with a proven (*A. phagocytophilum*) or potential (*A. platys* and *E. canis*) zoonotic role (Breitschwerdt et al. 2014; Sainz et al. 2015; Saito and Walker 2016) and have shown increasing prevalence worldwide in recent decades (Sainz et al. 2015). These microorganisms belong to the family Anaplasmataceae, order Rickettsiales, and are obligate intracellular bacteria, whose cycle in the environment involves complex interactions between invertebrate vectors and vertebrate hosts (Granick et al. 2023; Harrus et al. 2023). Indeed, numerous wild animal species are considered reservoir hosts (Ebani et al. 2008; Maia et al. 2014; Stuen et al. 2013; Torina et al. 2013; Pereira et al. 2016; Carvalho et al. 2017).

The geographical distribution of Anaplasmataceae species is related to tick survival and consequently to the temperature and the humidity of the area. They are widespread in states with temperate climates, usually with temperatures between 10 and 30 °C (Kidd 2019¸ El Hamiani Khatat et al. 2021). *A. platys* and, after the first recent detection in Australia, *E. canis* are now present in all continents (Sykes 2014; Carvalho et al. 2017; Neave et al. 2022), while for *A. phagocytophilum* Oceania is the only continent where no cases have yet been reported (Sykes and Foley 2014). In Italy, the presence of members of the Anaplasmataceae family is known from screening studies performed on dogs; however, results are extremely variable in relation to the diagnostic test used, geographical area investigated and inclusion criteria adopted (Petruccelli et al. 2021; Piantedosi et al. 2017). In Italy, *A. phagocytophilum* seroprevalence in dogs is estimated between 29% and 41.5% and molecular prevalence between 0% and 5.1%, but there are discordant studies on positivity in the different Italian regions (Solano-Gallego et al. 2006; Trotta et al. 2009; Mendoza-Roldan et al. 2021). There are few studies about the prevalence of *A. platys* in Italy, Trotta and colleagues estimated a molecular prevalence of 3.7% in dogs (2009), while a recent study estimated a molecular prevalence of 0.8% in dog-ticks (Zanet et al. 2020). Moreover, the true prevalence of these two pathogens is questioned due to a possible cross-reactivity between *Anaplasma* species, which cannot be discriminated by serological tests (Petruccelli et al. 2021). *E. canis*, on the other hand, shows a seroprevalence between 28.7% and 44%, while molecular positivity varies between 2.9% and 9.7% with the highest percentages detected in dogs in southern regions (Solano-Gallego et al. 2006; Mendoza-Roldan et al. 2021).

In dogs, the clinical signs related to these pathogens are often similar but highly variable depending on the virulence of the strain, the host’s immune response and the presence of coinfections or comorbidities (Sykes 2014). Nevertheless, granulocytic anaplasmosis is usually considered an acute disease with the development of non-specific clinical signs such as fever, lethargy, anorexia, and sometimes musculoskeletal pain and lameness (Greig et al. 1996; Granick et al. 2023). Ecchymosis, petechiae and gastrointestinal signs are rarely reported (Kohn et al. 2008). Differently, CME potentially recognise three clinical phases: an acute stage with non-specific signs and sometimes bleeding (petechiae, ecchymosis, epistaxis) (Sainz et al. 2015; Harrus et al. 2023), a subclinical stage without evident signs (Mylonakis et al. 2019), and a chronic stage characterised by the same, but more severe, clinical signs of the acute stage (Harrus et al. 2011). In the latter stage, neurological impairment, ocular lesions, ulcerative stomatitis and glomerulonephritis may be observed (Sainz et al. 2015; Mylonakis et al. 2019; Ziliani et al. 2019). Thrombocytopenia, anaemia and hyperglobulinaemia and proteinuria are the most common laboratory findings associated with *Anaplasma* and *Ehrlichia* infection (Poitout et al. 2005; Sainz et al. 2015).

A confirmed diagnosis of these diseases is particularly complex because of the frequent subclinical course of the disease itself or its manifestation with non-specific clinical signs (Clark et al. 1996). Currently, the diagnosis of Anaplasmataceae species infection in dogs is achieved by the combined use of different methods: (i) cytologic examination of Romanowsky-stained peripheral blood and buffy-coat smear, as well as lymph nodes and spleen aspirates, evidencing intracytoplasmic inclusion bodies (morulae) in granulocytes, mononuclear cells or platelets (Olano and Aguero-Rosenfeld 2008; Harrus and Waner 2011); (ii) rapid in-clinic immunoenzymatic (enzyme-linked immunosorbent assay, ELISA) tests validated to detect specific antibodies in serum samples (Little et al. 2014; Liu et al. 2018); (iii) serologic assays as indirect fluorescent antibody test (IFAT) and ELISA (Neer et al. 2002); and (iv) molecular assays able to detect pathogen DNA mainly in blood samples (Sainz et al. 2015). All the diagnostic tests have strengths and limitations in the different stage of infection (Allison and Little 2013; Diniz and Moura de Aguiar 2022). Direct tests show a better performance in the acute phase of infection because morulae and pathogen DNA are readily detected in whole blood samples (Harrus et al. 1998). Indirect testing is more effective in the later stages of infection, as antibodies against pathogens can take several weeks to become detectable after exposure, or when antibiotics have been administered before diagnosis, making direct testing ineffective (Egenvall et al. 2000).

The primary aim of this study was to assess the frequency of *Anaplasma* spp. and *Ehrlichia* spp. exposure in dogs tested by different diagnostic assays in a veterinary teaching hospital (VTH) in Italy. Secondary aims were to compare the performance of the different tests used, to evaluate correlations between infection and clinical data, and to genetically analyse the identified bacteria.

## Materials and methods

### Study design and inclusion criteria

For the purposes of the study, dogs referred to VTH of the University of Bologna (Italy) between 2012 and 2020 and tested with at least one among rapid immunoenzymatic test (RIT), IFAT or end-point PCR assay (PCR) for *Anaplasma* spp. or *Ehrlichia* spp. detection, were retrospectively included. Dogs were tested if they had clinical signs or clinicopathological alteration or risk factors related to infection, and if they were potential blood-donor animals. RIT tests were carried out in accordance with the manufacturer’s recommendation and IFAT and PCR tests were carried out on fresh material or samples stored at − 20 °C for a maximum of seven days until examination. All laboratory tests were performed no later than 2020. The study was carried out only on data retrieved from medical records and sequencing of PCR products obtained for diagnostic purposes following owner’s informed consent. No sampling or analyses were carried out for the purpose of this study and no experimental animals were involved.

Year of sampling and signalment data (sex, age, breed and geographical origin) of the enrolled dogs were retrieved from medical records. Dogs included were grouped according to the four different age categories proposed by Harvey (2021): (i) puppy, juvenile and young adult dogs (≤ 24 months); (ii) mature adult dogs (25–84 months); (iii) senior adult dogs (85–156 months); and (iv) geriatric dogs (> 156 months). Purebred dogs were divided according to the size, attitude and possible risk factors in: (i) toy breeds group, (ii) hunting breeds group, (iii) shepherd and guardian breeds group, and (iv) “other” breeds group when the dogs did not fit into any of the previous classes. Furthermore, the study population was divided in three groups according to the geographical area of origin in: Northern Italy (including dogs from Emilia-Romagna, Friuli Venezia Giulia, Liguria, Lombardia, Piemonte, Trentino Alto Adige, Valle d’Aosta, and Veneto regions), Central Italy (including dogs from Abruzzo, Lazio, Marche, Toscana, and Umbria regions) and Southern Italy (including dogs from Puglia, Basilicata, Calabria, Campania, Molise, Sardegna, and Sicilia regions) groups. In dogs tested positive, clinical and clinicopathological data including complete blood count (CBC), serum biochemistry and urine protein to creatinine ratio (UPC) were retrieved from medical records (Online Resource 1).

### Diagnosis of infection by rapid immunoenzymatic, serological and molecular tests

A RIT for the simultaneous detection of *Dirofilaria immitis* antigen and antibodies against *A. phagocytophilum* and *A. platys*, *E. canis* and *E. ewingii*, and *Borrelia burgdorferi* (SNAP 4DX, IDEXX Laboratories, Westbrook, ME, USA) was carried out on blood or serum specimens, following the manufacturer’s instructions.

*Anaplasma phagocytophilum* and *E. canis* IFAT for IgG detection (MegaFLUO ANAPLASMA phagocytophilum and MegaFLUO EHRLICHIA canis, MEGACOR Diagnostik, Horbranz, Austria) were performed on serum samples, following the manufacturer’s instructions. Briefly, slides coated with *A. phagocytophilum* and *E. canis* infected cells were probed with sera serially diluted in phosphate-buffered saline (PBS) starting with a concentration of 1:40 until reaching a concentration of 1:1280, incubated at 37 °C for 30 min and washed two times with PBS. Internal canine positive and negative sera controls were included on each slide. The slides were probed with 20 μL of fluorescein isothiocyanate (FITC) conjugated anti-dog IgG antibody diluted in PBS at a concentration of 1:64 (Anti-Dog IgG-FITC antibody produced in rabbit; Sigma-Aldrich, Saint Louis, MO, USA) at 37 °C for 30 min and were washed two times with PBS and examined under a fluorescent microscope. The highest dilution showing fluorescence was the final antibody titre. Samples that showed no fluorescence were considered negative.

DNA was extracted from blood samples using the NucleoSpin Tissue Kit (Macherey-Nagel, Düren, Germany), according to the manufacturer’s instructions. The extracted DNA were stored at − 20°C until use. The detection of all known *Anaplasma* spp. and *Ehrlichia* spp. DNA was carried out with a previously described end-point PCR assay (Balboni et al. 2021) using the Taq DNA Polymerase Kit (Qiagen, Hilden, Germany), according to the manufacturer’s instructions. An internal positive control and a no template control, consisting of ultrapure water, underwent analysis simultaneously. A fragment of about 600 bp of the groEL gene of *Anaplasma* spp. and *Ehrlichia* spp. was amplified using the primers groEL_For (5’-ACT GAT GGT ATG CAR TTT GAY CG-3’) and groEL_Rev (5’-TCT TTR CGT TCY TTM ACY TCA ACT TC-3’) (Barber et al. 2010). The thermal cycling consisted of an initial denaturation at 94 °C for 5 min followed by 45 cycles of denaturation at 94 °C for 30 s, annealing at 58 °C for 30 s and elongation at 72 °C for 45 s, followed by a final elongation step at 72 °C for 10 min. PCR products were visualized under UV after electrophoresis migration on a 1.5% agarose gel stained with ethidium bromide or Midori Green Advance DNA Stain (Nippon Genetics, Düren, Germany) in 1X standard tris-acetate-EDTA (TAE) buffer. Amplicons of the expected size were considered positive.

Dogs tested positive for more than one pathogen with the tests used were considered potentially coinfected.

### Sequence analysis

Amplicons of the expected size were purified using the QIAquick PCR Purification Kit (Qiagen, Hilden, Germany) according to the manufacturer’s instructions and directly sequenced by Sanger method (BioFab Research, Italy) using both forward and reverse primers. The nucleotide sequences obtained were assembled and translated into amino acid sequences to evaluate their correct translation using BioEdit sequence alignment editor version 7.2.5. The assembled nucleotide sequences were analysed using the BLAST web interface to determine which species they belonged to (https://blast.ncbi.nlm.nih.gov/Blast.cgi) and aligned with 55 *A. phagocytophilum*, 17 *A. platys*, eight *A. platys-*like and nine *E. canis* reference sequences available in the GenBank database (https://www.ncbi.nlm.nih.gov/nucleotide/), using the ClustalW method implemented in the BioEdit software. Phylogeny was carried out with the MEGA 11 software version 11.0.11 (Tamura et al. 2021) using Maximum Likelihood method and the Tamura 3-parameter model. The robustness of individual nodes on the phylogenetic tree was estimated using 1,000 bootstrap replicates and relevant bootstrap values were indicated at the corresponding node.

### Statistical analysis

All the collected data were captured in Microsoft Excel 2019 and analysed using a commercially available statistical software (MedCalc Statistical Software version 19.5.1, Ostend, Belgium). The Shapiro-Wilk test was used to assess the distribution of continuous variables. Descriptive statistics was performed for all the evaluated variables and data are reported as mean ± standard deviation or median and range (minimum-maximum values), based on their distribution. Categorical data, such as year of sampling, sex, age and breed categories, and geographic origin, were analysed using the Fisher’s exact *P*-value test or Pearson’s chi-squared (χ2) test. Continuous data (e.g. clinicopathological findings) were analysed using the Mann-Whitney or Kruskal-Wallis tests. Inter-rater agreement test (Cohen’s kappa coefficient) was calculated to compare the results obtained by RIT, IFAT and PCR in dogs tested with two or more assays. Kappa values ≤ 0 were interpreted as indicating no agreement, 0.01–0.20 as no to mild agreement, 0.21–0.40 as fair agreement, 0.41–0.60 as moderate agreement, 0.61–0.80 as substantial agreement and 0.81–1.00 as near-perfect agreement between the compared tests. A *P*-value < 0.05 was considered significant.

## Results

### Study population

One thousand three hundred twenty-two dogs were tested with at least one assay for *Anaplasma* spp. or *Ehrlichia* spp. detection and were included in the study. Year of sampling, signalment data and clinicopathological findings of the enrolled dogs are reported in Table 1. The number of dogs tested increased during the years: 50/1322 (3.8%) dogs were tested in the first year of the study (2012), an initial peak was reached in 2014 (155/1322, 11.7% dogs) followed by a decrease in the following two years (2015 and 2016) and then a progressive increase up to 216 dogs tested was reported in each of the last two years of the study (2019 and 2020). As potential blood-donor animals, 102/1322 (7.7%) apparently healthy dogs were tested, all in the last four years (2017–2020). Among the dogs included, 657/1322 (49.7%) were male (130/657, 19.8% castrated) and 665/1322 (50.3%) were female (356/665, 53.5% spayed). The median age was 5 years and 7 months (range 1 month − 17 years). For four dogs the age was not available. In particular, most of the dogs were mature or senior adults: 542/1318 (41.1%) and 445/1318 (33.8%), respectively. Among the dogs included, 482/1322 (36.5%) were mixed breed and 840/1322 (63.5%) purebreds, with 93 different breeds reported. Based on the above reported categories 137/840 (16.3%) were toy dogs, 199/840 (23.7%) hunting dogs, 234/840 (27.9%) shepherd and guardian dogs, and 270/840 (32.1%) were dogs of other breeds. Most of the dogs (1232/1322, 93.2%) were from Northern Italy, whereas 57/1322 (4.5%) and 33/1322 (2.5%) were from Central and Southern Italy, respectively.

**Table 1:** Signalment data and year of sampling of dogs tested for *Anaplasma* spp. and *Ehrlichia* spp. exposure

Variables	Total	Total positive	*P* value	Tested for A. ph	Positive to A. ph	*P* value	Tested for A. pl	Positive to A. pl	*P* value	Tested for E. ca.	Positive to E. ca.	*P* value	Tested for A. ph and E. ca.	Positive to A. ph and E. ca.	*P* value	
Number of dogs	1322	94 (7.1%) *^2^		1246	53 (4.3%)		920	1 (0.1%)		1312	63 (4.8%)		1237	24 (1.9%)		
Year of sampling			**0.0041**			**0.0001**			0.2880			0.2619			0.2105	
	2012	50	6 (12%)		41	4 (9.8%)		1	0 (0%)		50	2 (4.0%)		41	0 (0%)	
	2013	129	11 (8.5%)		109	8 (7.3%)		81	0 (0%)		127	6 (4.7%)		107	3 (2.8%)	
	2014	155	20 (12.9%)		142	13 (9.2%)		76	0 (0%)		151	12 (7.9%)		138	5 (3.6%)	
	2015	97	6 (6.2%)		89	4 (4.5%)		22	0 (0%)		95	4 (4.2%)		87	2 (2.3%)	
	2016	93	10 (10.8%)		82	5 (6.1%)		26	0 (0%)		93	8 (8.6%)		82	3 (3.7%)	
	2017	188	7 (3.7%)		182	4 (2.2%)		144	0 (0%)		188	4 (2.1%)		182	1 (0.5%)	
	2018	178	8 (4.5%)		173	4 (2.3%)		154	0 (0%)		177	8 (4.5%)		172	4 (2.3%)	
	2019	216	15 (6.9%)		214	6 (2.6%)		207	0 (0%)		215	12 (5.6%)		214	4 (1.9%)	
	2020	216	11 (5.1%)		214	5 (2.3%)		209	1 (0.5%)		216	7 (3.2%)		214	2 (0.9%)	
Sex			**0.0486**			0.1990			0.9844			0.0787			0.5875	
	Male	657	37 (5.6%)		613	21 (3.4%)		451	1 (0.2%)		652	24 (3.7%)		609	10 (1.6%)	
	Female	665	57 (8.6%)		633	32 (5.1%)		469	0 (0%)		660	39 (5.9%)		628	14 (2.2%)	
Age (months) *^1^	67 (1-212)	82 (2-194)	0.4645	68 (1-212)	85 (8-194)	0.0794	76 (1-212)	10		67 (1-212)	71 (2-194)	0.7671	68 (1-212)	78 (8-194)	0.5397	
Age groups			0.1611			**0.0299**			0.1709			0.9502			0.5328	
	Puppy, juvenile and young adult dogs	247	14 (5.7%)		231	6 (1.7%)		153	1 (0.7%)		246	11 (4.5%)		230	2 (0.9%)	
	Mature adult dogs	542	35 (6.5%)		507	22 (4.3%)		349	0 (0%)		537	25 (4.7%)		502	12 (2.4%)	
	Senior adult dogs	445	40 (9.0%)		426	26 (6.1%)		346	0 (0%)		441	22 (5.0%)		423	9 (2.1%)	
	Geriatric dogs	84	3 (3.6%)		80	1 (1.3%)		71	0 (0%)		84	3 (3.6%)		80	1 (1.3%)	
Breed			**< 0.0001**			0.2925			0.7850			**< 0.0001**			**0.0034**	
	Mixed breed	482	56 (11.6%)		450	24 (5.3%)		339	1 (0.3%)		479	47 (9.8%)		447	16 (3.6%)	
	Purebred	840	38 (4.5%)		796	29 (3.6%)		581	0 (0%)		833	16 (1.9%)		790	8 (1.0%)	
Purebred groups			0.0847			**0.0156**						0.6695			0.0974	
	Toy breeds	137	4 (2.9%)		131	3 (2.3%)		112	0 (0%)		137	3 (2.2%)		131	2 (1.5%)	
	Hunting breeds	199	14 (7.0%)		190	14 (7.4%)		150	0 (0%)		196	4 (2.0%)		187	4 (2.1%)	
	Shepherd and guardian breeds	234	13 (5.6%)		220	7 (3.2%)		152	0 (0%)		231	6 (2.6%)		218	1 (0.5%)	
	Other breeds	270	7 (2.6%)		255	5 (2.0%)		167	0 (0%)		269	3 (1.1%)		254	1 (0.4%)	
Geographical origin			0.8617			0.9319			**< 0.0001**			0.8756			0.7280	
	Northern Italy group	1232	87 (7.1%)		1160	50 (4.3%)		859	0 (0%)		1233	59 (4.8%)		1152	23 (2.0%)	
	Central Italy group	57	5 (8.8%)		55	2 (3.6%)		42	1 (2.4%)		56	3 (5.4%)		54	1 (1.9%)	
	Southern Italy group	33	2 (6.1%)		31	1 (3.2%)		19	0 (0%)		33	1 (3.0%)		31	0 (0%)	

*A. ph: Anaplasma phagocytophilum; A. pl: Anaplasma platys; E. ca.: Ehrlichia canis*

*^1^ Data are reported as median and (range minimum-maximum). *^2^ One dog tested positive was excluded from the statistical analyses referring to the different pathogens investigated because it had tested positive only by PCR for all known Anaplasmataceae but its nucleotide sequence had not obtained for characterisation

### Diagnosis of infection by rapid immunoenzymatic, serological and molecular tests

**Fig. 1:**
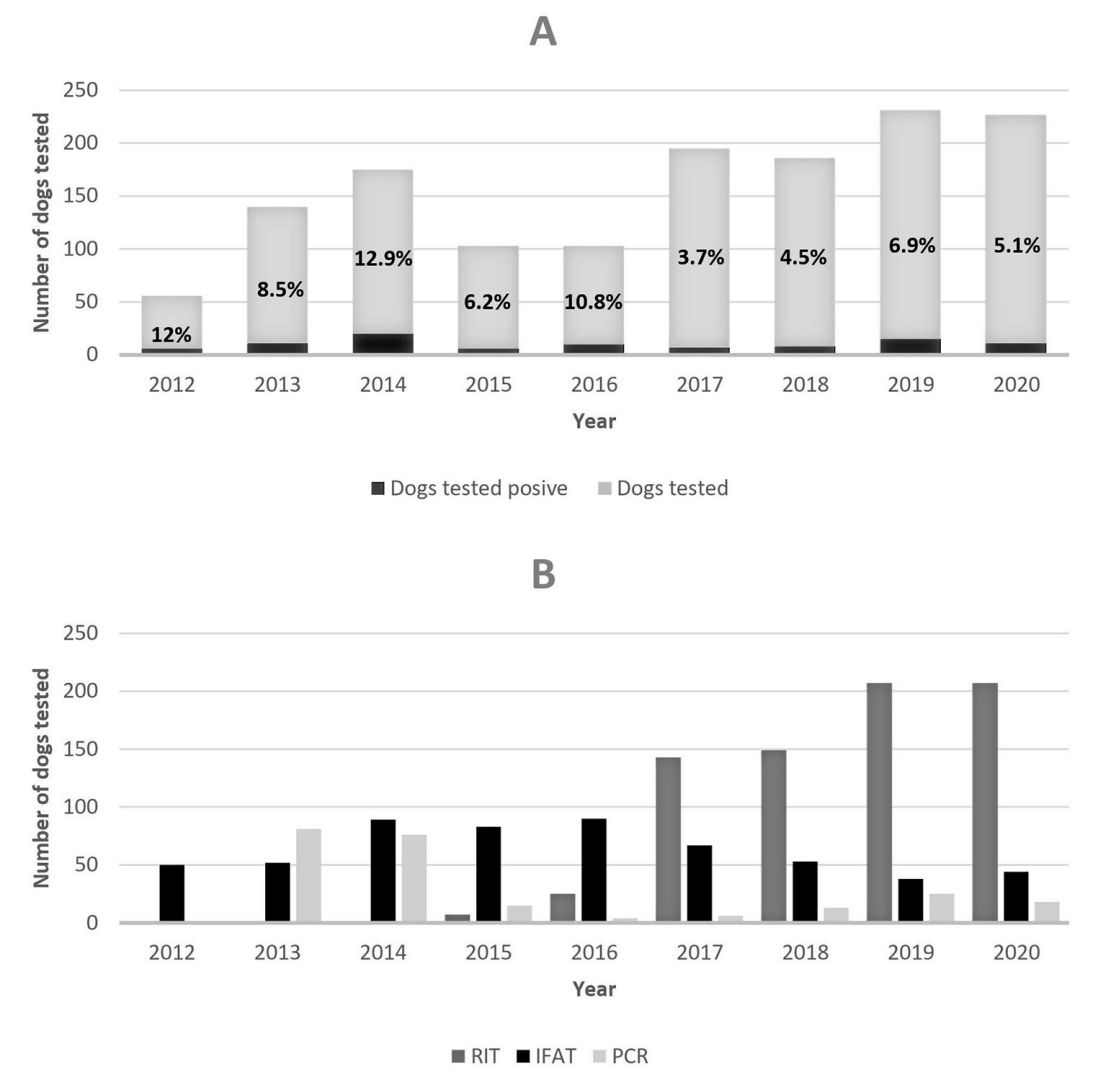
Dogs tested positive (**A**) and type of test used (**B**) during the study period A) highlighted in bold: percentages of positives on number of dogs tested per year B) IFAT: indirect fluorescent antibody test; NT: not tested; PCR: polymerase chain reaction, RIT: rapid immunoenzymatic test

Ninety-four of the 1322 (7.1%) dogs tested positive for at least one pathogen investigated in the study. The frequency of dogs tested positive had fluctuations but it significantly decreased over the study period (*P* = 0.0041, Table 1; Fig. 1). All the 102 potential blood-donor dogs tested negative and even excluding them the positivity rate significantly decreased over the study period (*P* = 0.019).

A significant association was found between positive result to at least one pathogen investigated and the sex of dogs, with females (57/665, 8.6%) more frequently positive than males (37/657, 5.6%) (*P* = 0.0486). The median age of dogs tested positive was 6 years and 10 months (range 2 months − 16 years) with no significance difference between age categories. The frequency of positive dogs was significantly higher in mixed breed (56/482, 11.6%) than in purebred (38/840, 4.5%) dogs (*P* < 0.0001). Differently, no significant association was evidenced between the four breed groups: toy breeds, hunting breeds, shepherd and guardian breeds, and other breeds. No other significant association was found between the frequency of infection and signalment data analysed (Table 1), including the geographical origin of the dogs tested positive, which appears equally distributed between Northern (87/1232, 7.1%), Central (5/57, 8.8%) and Southern (2/33, 6.1%) Italy. Clinical signs and clinicopathological variables of the dogs tested positive had no significant association with the presence of infections with the different pathogens considered in the study (Online Resources 2, 3 and 4). An exception was proteinuria, assessed by UPC values for 58 of 94 (61.7%) dogs tested positive. Indeed, of the 26/58 (44.8%) proteinuric dogs, those tested positive for *A. phagocytophilum* presented proteinuria more frequently compared to those tested positive for the other pathogens (*P* = 0.0487).

Fifty-three of the 1246 (4.3%) dogs tested for *A. phagocytophilum* were positive. The frequency of dogs tested positive for *A. phagocytophilum* significantly decreased over the study period (*P* < 0.0001, Table 1; Fig. 1), with 34/463 (7.3%) dogs tested positive in the first five years (2012–2016) and 19/783 (2.4%) dogs tested positive in the last four years (2017–2020). A slightly significant higher frequency of *A. phagocytophilum* detection was found in senior adult (26/426, 6.1%) dogs compared to other age groups (*P* = 0.0299), and in hunting breed dogs (14/190, 7.4%) compared to the other breed groups (*P* = 0.0156). No other significant association was found between *A. phagocytophilum* infection and signalment data analysed (Table 1).

One of the 920 (0.1%) dogs tested for *A. platys* was positive. It was a mixed-breed male dog of ten months old from a kennel in Rome (Central Italy) in 2020 (Table 1; Fig. 1).

Sixty-three of the 1312 (4.8%) dogs tested for *E. canis* were positive. The frequency of dogs tested positive for *E. canis* showed a constant distribution over the study period (Table 1; Fig. 1), whereas this frequency was significantly higher in mixed breed (47/479, 9.8%) than in purebred (16/833, 1.9%) dogs (*P* < 0.0001). No other significant association was found between *E. canis* infection and the signalment data analysed (Table 1).

Twenty-four of the 1237 (2.1%) dogs tested for *A. phagocytophilum* and *E. canis* were positive to both pathogens, evidencing a state of potential coinfection. No significant association was found between potential coinfection and the signalment data analysed, with the exception of a higher frequency of potential coinfection in mixed breed (16/447, 3.6%) than in purebred (8/790, 1.0%) dogs (*P* = 0.0034, Table 1).

One dog that tested positive only in PCR was excluded from the statistical analysis regarding the specific pathogens investigated because its nucleotide sequence was not obtained for the bacterial species identification.

During the study period 1544 tests were carried out: 739 (47.9%) RIT, 566 (36.7%) IFAT and 239 (15.5%) PCR. IFAT was used since the start of the study period, with an increasing number of tests carried out in the first years and a progressive decrease starting from 2017. PCR was introduced into the diagnostic routine since 2013, with a large number of tests carried out in the first two years, followed by significantly lower and constant numbers since 2015. RIT was introduced into the diagnostic routine since 2015 and the number of tests carried out progressively increased until 2020 (Fig. 1). The distribution of positive results according to the tests used was: 38/739 (5.1%) for RIT, 79/566 (14.0%) for IFAT and 15/239 (6.3%) for PCR. Several dogs were tested by more than one type of assay: 112/1322 (8.5%) were tested with RIT and IFAT, 24/1322 (1.8%) with RIT and PCR, 20/1322 (1.5%) with IFAT and PCR, and 33/1322 (2.5%) with all three assays. Of the 94 dogs tested positive, 45 were tested by RIT and 38/45 (84.4%) were positive, 82 were tested by IFAT and 79/82 (96.3%) were positive, and 39 were tested by PCR and 15/39 (38.5%) were positive (Online Resource 5). In Table 2 (**A**, **B** and **C**), the results obtained with the different tests used were reported and compared. In particular, 145 dogs were tested with both IFAT and RIT, 57 with both RIT and PCR, and 53 with both IFAT and PCR. The values of Cohen’s kappa coefficient were consistent with a near-perfect agreement between RIT and IFAT (0.88) and a poor agreement between PCR and RIT (0.21) or PCR and IFAT (0.17).

**Table 2:** Comparison between tests results in dogs positive for at least one pathogen

		Pos	Neg	Total
**A**	**IFAT**			
**RIT**	Pos	29 (20.0%)	4 (2.8%)	33 (22.8%)
	Neg	2 (1.4%)	110 (75.9%)	112 (77.2%)
	Total	31 (21.4%)	114 (78.6%)	145 (100%)
**B**	**PCR**			
**RIT**	Pos	6 (10.5%)	16 (28.1%)	22 (38.6%)
	Neg	3 (5.3%)	32 (56.1%)	35 (61.4%)
	Total	9 (15.8%)	48 (84.2%)	57 (100%)
**C**	**IFAT**			
**PCR**	Pos	7 (13.2%)	1 (1.9%)	8 (15.1%)
	Neg	23 (43.4%)	22 (41.5%)	45 (84.9%)
	Total	30 (56.6%)	23 (43.4%)	53 (100%)

A: Comparison between RIT and IFAT test results

B: Comparison between RIT and PCR test results

C: Comparison between PCR and IFAT test results

IFAT: indirect fluorescent antibody test; Neg: negative result; PCR: polymerase chain reaction; Pos: positive result; RIT: rapid immunoenzymatic test

### Sequence analysis

Partial groEL gene nucleotide sequences of *Anaplasma* spp. or *Ehrlichia* spp. were obtained for 13/15 PCR positive dogs: on the basis of BLAST analysis, four were *A. phagocytophilum*, one was *A. platys* and eight were *E. canis*. In two dogs, nucleotide sequences were not obtained due to the low amount of PCR product: the first was PCR-positive only and it was excluded from the specific statistical analyses; the second one was tested positive by both RIT and IFAT tests for *A. phagocytophilum* and was considered infected with the latter. The sequences of *A. phagocytophilum* obtained in this study (lab and GenBank IDs: 393/2012 KF778380, 862/2014 KT970678, 901/2014 KT970679, and 909/2014 KT970680, the first three sequences were previously reported in Dondi et al. 2014 and De Arcangeli et al. 2018) showed a nucleotide identity of 99.5–100% among them. The only *A. platys* detected was sequenced in the dog 682/2020 (GenBank ID: OR209783) and IFAT and SNAP tests identified it as *E. canis*. This *A. platys* sequence showed a nucleotide identity of 99.7–100%, 71.9% and ≤ 77.9% with *A. platys*, *E. canis* and *A. phagocytophilum* reference sequences, respectively. The sequences of *E. canis* obtained in this study (lab and GenBank IDs: 598/2014 OR209777, 810/2014 OR209778, 1382/2016 OR209779, 421/2019 OR209780, 438/2019 OR209781, 501/2019 OR209782, 636/2019 ON245149, 637/2019 ON245150, the last two sequences were previously reported in Urbani et al. 2022) were identical.

**Fig. 2:**
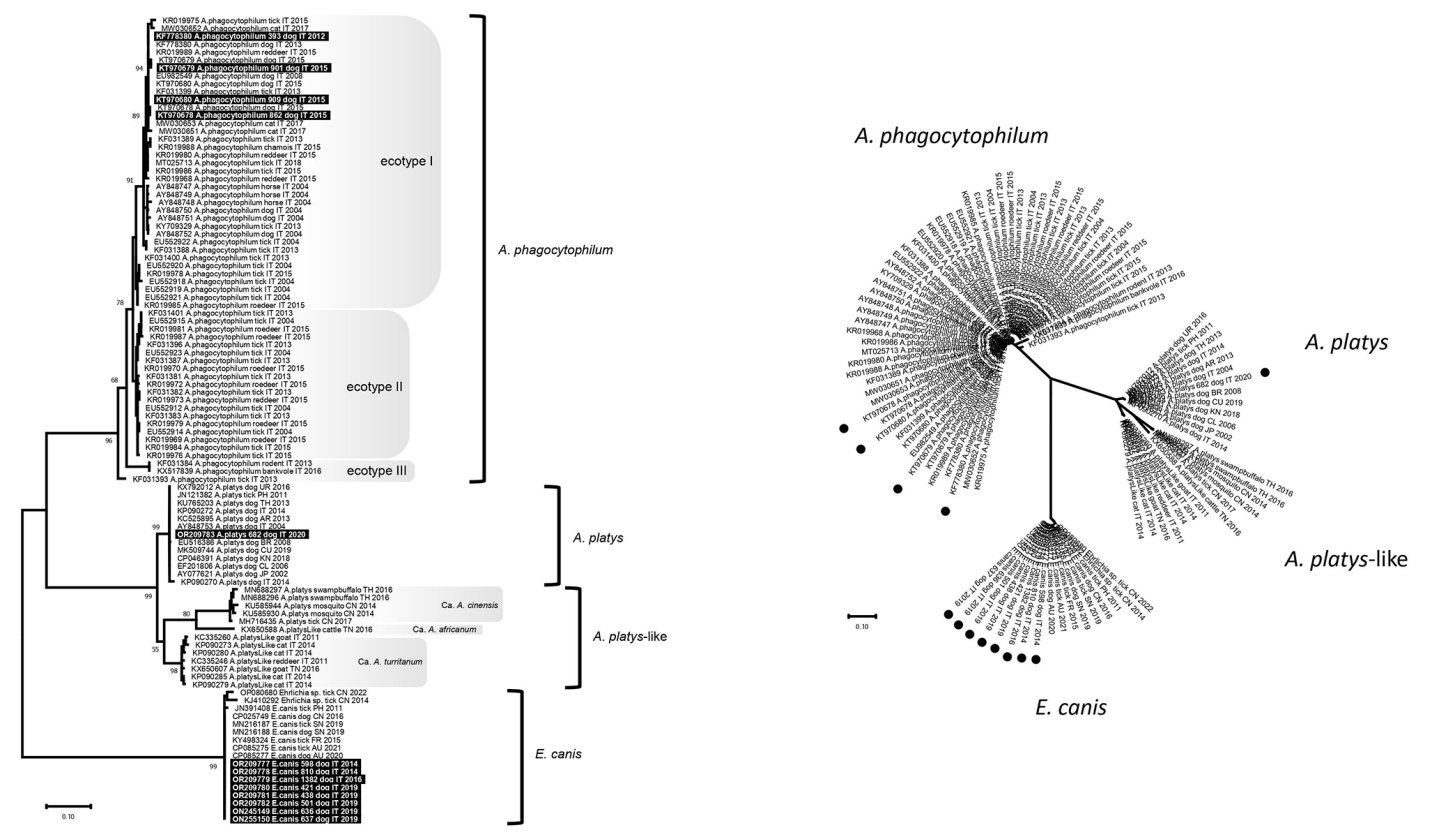
Phylogenetic tree based on partial heat shock gene (groEL) nucleotide sequences of *Anaplasma phagocytophilum*, *A. platys* and *Ehrlichia canis*. Phylogeny was evaluated using the Maximum Likelihood method and the Tamura 3-parameter model implemented on MEGA 11 software version 11.0.11 on multiple alignment constructed with nucleotide sequences obtained in this study, 55 reference sequences of *A. phagocytophilum*, 17 of *A. platys*, 8 of *A. platys-*like and 9 of *E. canis* retrieved from GenBank. On the left a traditional rectangular branch style of the tree and on the right a radiation branch style of the tree. Identification of the sequences undergoes the following nomenclature: GenBank accession number, species, strain (only for sequences obtained in this study), host, country (AR: Argentina, AU: Australia, BR: Brazil, CL: Chile, CN: China, CU: Cuba, FR: France, IT: Italy, JP: Japan, KN: Saint Kitts and Navis, PH: Philippines, SN: Senegal, TH: Thailand, TN: Tunisia, UR: Uruguay), and collection date (or date of database submission). Statistical support was provided by bootstrapping with 1000 replicates and values indicated on the respective branches. Highlighted in black or black circles: sequences generated in this study. Main clusters of *A. phagocytophilum* are labelled following the ecotype classification proposed by Jahfari and colleagues (Jahfari et al. 2014)

The phylogenetic tree showed four main clusters, corresponding to the species of pathogen investigated (Fig. 2).

In the *A. phagocytophilum* cluster, composed by sequences from Italy, three subgroups corresponding to three ecotypes proposed by Jahfari and colleagues (2014) were evidenced. The *A. phagocytophilum* sequenced in this study clustered in the ecotype I subgroup with other sequences identified in ticks and several mammalian hosts (dog, horse, red deer and chamois). The nucleotide sequence 682/2020 grouped in the *A*. *platys* cluster with reference strains from different geographical areas of the world. The so-called *A. platys-*like cluster had three subgroups composed by *A*. *cinensis*, *A. africanum* and *A. turritanum* candidates, respectively (Zobba et al. 2022a, b). Nucleotide sequences of *E. canis* obtained in this study grouped in a single cluster with the *E. canis* reference strains identified in ticks and dogs from various countries.

## Discussion

This study was primarily aimed to describe the frequency of *Anaplasma* spp. and *Ehrlichia* spp. exposure in a veterinary hospital population of dogs in Italy, retrospectively enrolled in a 9-year period from 2012 to 2020. Performance of the tests adopted, correlations between infection and clinical data, and phylogeny of the identified bacteria were also investigated. A total of 94/1322 dogs tested positive to *Anaplasma* spp. or *Ehrlichia* spp. antibodies or DNA, with an overall frequency of infection of 7.1%. In particular, the values of frequency of infection of *A. phagocytophilum* (53/1246, 4.3%), *A. platys* (1/920, 0.1%), and *E. canis* (63/1312, 4.8%) obtained in this study were lower compared with previous surveys carried out in Italy which, however, were highly variable in relation to the diagnostic tests used, geographical area investigated and inclusion criteria adopted (Piantedosi et al. 2017; Petruccelli et al. 2021). In fact, some authors reported seroprevalence in dogs between 29% and 41.5% for *A. phagocytophilum* and between 28.7% and 44% for *E. canis* (Trotta et al. 2009; Mendoza-Roldan et al. 2021), while other surveys reported molecular prevalence in dogs between 0% and 5.1% for *A. phagocytophilum*, 0.8% and 3.7% for *A. platys*, and between 2.9% and 9.7% for *E. canis* (Solano-Gallego et al. 2006; Trotta et al. 2009; Zanet et al. 2020).

During the study period, the frequency of exposure detected decreased significantly, with highest frequency in 2012 (6/50, 12%) and 2014 (20/155, 12.9%) and a progressive decline from 2016 to 2020 (11/216, 5.1%). This trend was predominantly associated with the number of dogs tested positive to *A. phagocytophilum* and could be related to the climatic conditions, that are known to strongly influence the presence of ticks in the environment and consequently the spreading of vector-borne pathogens (Ebani 2019). Indeed, the year 2014, which showed the highest frequency of positivity detected, was a very warm and rainy year in Italy, with the average annual temperature 1.63 °C higher than the normal value and a total annual rainfall overall higher of about 13% than the climatic average (Desiato et al. 2014). Furthermore, the progressive decrease in frequency of positivity detected could be linked to the increasing use of ectoparasiticides by dog-owners or to several factors which led to carrying out a greater number of tests for screening purposes, testing animals at risk of infection and not only those with associated clinical signs, therefore with a lower probability of testing positive: (i) the introduction into the diagnostic routine of a rapid and easy-to-use test such as the RIT (since 2015), which has also led to a reduction in the use of IFAT and PCR; (ii) the increasing sensitivity of veterinarians to these pathogens, given the increasing prevalence of the associated diseases; and (iii) the launch in 2017 of our veterinary blood bank (Veterinary Blood Solutions – VeBS) in the VTH with routine tests of blood donor dogs with low suspicion of infection. Consistently, Morganti and colleagues (2022) reported a lower prevalence of infection in potential blood donor dogs compared to a non-selected dog population.

Several studies reported that sex is not a risk factor for *Anaplasma* spp. and *Ehrlichia* spp. infection or reported a slight predisposition in male dogs, probably related to their behaviour which exposes them more to ticks (Sainz et al. 2015; Ebani 2019; Hazelrig et al. 2023). Differently, in our study a slight predisposition to infection was found in female dogs, a result probably linked to random sampling. In our study, moreover, a slight association between dog age and infection was found for *A. phagocytophilum* only, with senior adult dogs (6–10 years of age) being more predisposed to contract the infection, a finding consistent with other studies (Watanabe et al. 2004; Costa et al. 2007; Ebani et al. 2013). This predisposition is probably due to an increased risk of exposure of the senior adult dogs to TBP over time, rather than an increased susceptibility to them (Egenvall et al. 2000; Kohn et al. 2011). Some authors reported a higher prevalence of *Ehrlichia* spp. infection in adult dogs (Ebani 2019; Hazelrig et al. 2023), while in several studies age was not a risk factor for any of the TBP considered (Ebani et al. 2014; Sainz et al. 2015). A significantly higher prevalence of *A. phagocytophilum* infection was found in hunting breed dogs enrolled in this study, an association never reported in literature (Sainz et al. 2015; Piantedosi et al. 2017). On the other hand, some studies reported a link between TBP infection and the lifestyle or environments frequented by dogs of hunting breeds, due to closer contact with wooded and rural areas, cohabitation in outdoor kennels and potentially less consistent use of acaricide products. For these reasons, it is possible to speculate that hunting dogs have a predisposition to contracting the infection due to exposure to risk factors rather than to the breed (Kordick et al. 1999; Solano-Gallego et al. 2006; Piantedosi et al. 2017). Furthermore, *Ixodes ricinus* tick, vector of *A. phagocytophilum*, is widespread in environments and animals to which hunting dogs are more exposed than other dog breeds (Santoro et al. 2016; Westmoreland et al. 2016). Indeed, *I. ricinus* is found in mixed and deciduous forests, open pastures and other areas with high humidity and its wide distribution is also related to a broad host range, including many mammalian species and birds (Medlock et al. 2013). Differently, a predisposition to contract infection sustained by *E. canis* was found in this study for mixed-breed dogs compared to purebred ones. This finding is not in agreement with the literature which reports mixed-breed dogs as generally more resistant to infection (Harrus et al. 1997). As for hunting breed dogs, also the predisposition of mixed-breed dogs to contract TBP infection may be related to the lifestyle of the dogs investigated. Moreover, mixed-breed dogs are often adopted without knowing their infectious status or the epidemiological situation of the geographical area of origin, thus increasing the possibility that they were infected prior to adoption. Analysing the geographical origin of the dogs included in this study, the majority of enrolled dogs come from Northern Italy (1237) compared to other areas (57 and 33 dogs for Central and Southern Italy, respectively), as a result of the location of our veterinary hospital. Nevertheless, no significant differences in the frequency of exposure were found in our study in the different Italian geographical areas. Differently, several studies found significantly higher prevalence in Central and Southern Italian regions (Solano-Gallego et al. 2006; Vascellari et al. 2016; Colombo et al. 2021). This discrepancy in the results may be due to an underestimation of the prevalence of *Anaplasma* spp. and *Ehrlichia* spp. in Central and Southern Italy for a limited sampling of dogs from these regions. Proteinuria was the only clinicopathological variable that showed a significantly higher frequency in *A. phagocytophilum* infections compared to dogs tested positive for the other pathogens investigated in this study. This finding was already reported to be associated with this pathogen and this clinicopathological variable could be considered to differentiate the diagnosis of these TBP (Dondi et al. 2014; Ravnik et al. 2014).

The tests used in this study showed a different performance. The serological methods (RIT and IFAT) had high concordance among them and allowed the identification of a higher number of positive dogs compared to PCR. This result is in agreement with the characteristics of serological tests which, detecting antibodies against pathogens even after the infection has resolved, identify more positive dogs than the molecular methods. Conversely, PCR can only diagnose active infections and therefore identifies fewer positive dogs. In fact, molecular assays applied to blood samples are highly sensitive and specific but false-negative results may occur as consequence of pathogen load (Sainz et al. 2015), presence of inhibitors, variations in levels of circulating pathogens due to intermittent bacteraemia and antibiotics administration (Allison and Little 2013). Furthermore, *E. canis* often play a role in chronic infections which may not be easily identified by direct methods. For these reasons, negative results in molecular tests only indicate that the respective nucleic acid sequence is not detected in a particular sample at a particular point of time and should not be interpreted as conclusive evidence of absence of infection (Allison and Little 2013; Sainz et al. 2015; Diniz and Moura de Aguiar 2022). Furthermore, a major therapeutic dilemma is posed by asymptomatic dogs with positive tests results, because the treatment of choice against infections with bacteria of the Anaplasmataceae family is long-term antibiotic therapy. At a time when antimicrobial resistance and the reduction of the use of antimicrobial drugs are becoming increasingly important, it is difficult to assess the real need to treat positive dogs even in the absence of clinical or clinicopathological abnormalities (Harrus et al. 2023).

Phylogeny of the pathogens sequenced in this study showed a clear distinction between the three species examined. All the *A. phagocytophilum* obtained in this study clustered in the ecotype I subgroup (Jahfari et al. 2014; Jaarsma et al. 2019), which exhibits the widest host range and includes all strains identified in humans in Europe, suggesting potential zoonotic implications and reservoir role of wild animals (Balboni et al. 2021; Grassi et al. 2021). The *A. platys* obtained in this study clustered with other *A. platys* detected in dogs worldwide, revealing a high genetic similarity between strains from different geographical areas (de la Fuente et al. 2006; Zobba et al. 2015). All the *E. canis* obtained in this study clustered and showed an identity of 100% with reference strains identified in dogs and ticks from Europe, Asia and Africa, excluding correlation with host and geographical origin (Alhassan et al. 2021).

Of interest, the *A. platys* sequenced in this study was identified in a dog (682/2020) tested positive by RIT and IFAT, which were apparently indicative for *E. canis* exposure. This result can have two explanations. The dog could have antibodies against *E. canis* from a previous exposure and at the same time an active infection with *A. platys*, without detectable levels of specific antibodies. This type of coinfection has already been reported and may be related to sharing the same tick vector (*Rhipicephalus sanguineu*s, Piratae et al. 2019; Garcia Ribeiro et al. 2023). Otherwise, an antibody cross-reactivity between the two pathogens may have occurred, thus distorting the RIT and IFAT tests results. Few cases of cross-reactivity have been reported, but never between *E. canis* and *A. platys*. They often occur between different *Ehrlichia* species, or between *A. phagocytophilum* and *A. platys*, or even between *A. phagocytophilum* and *E. canis* (Al-Adhami et al. 2011; Qurollo et al. 2021). Further studies are needed to investigate the occurrence of potential cross-reactions between these two species.

The present study has some limitations due to its retrospective design. Firstly, the animals enrolled were not all tested with the three tests used, limiting the comparison between the results obtained and the evaluation of the sensitivity and specificity of the assays. Furthermore, only for 13/94 positive dogs the identified pathogen was typed by sequencing. In the other 81/94 positive dogs, the species identification was carried out on a serological basis, by RIT or IFAT, and could be subjected to errors, as demonstrated by the *A. platys* genetically identified in a dog with serological test results apparently indicative of *E. canis* exposure. Twenty-four of 94 (25.5%) dogs were considered potentially coinfected because tested positive for more than one pathogen. In dogs where multiple positivity was detected only by a serological test (RIT or IFAT) it was not possible to exclude with certainty (gene sequencing of the bacteria identified by PCR) that antibody cross-reactivity between different pathogens or serological positivities related to previous resolved infections have occurred, resulting in incorrect identification of the status of coinfection. Although sequencing of end-point PCR products is a very useful tool in single-pathogen infections, it alone is not conclusive in the correct identification of multiple pathogens during coinfections. Another limit was the geographical distribution of the enrolled dogs, mostly from Northern Italy, which was linked to our VTH location. Consequently, the dog population analysed does not adequately represent all of Italy, but allows to draw more reliable conclusions for dogs from Northern Italy and to compare them with the dogs moving from Central and Southern Italy to be visited in a veterinary hospital of reference in the North.

## Conclusion

Our study reports the trend over time of the dogs tested positive for *Anaplasma* spp. and *Ehrlichia* spp. according to the tests used and the population analysed and provides new insights into possible risk factors for infection in dogs in Italy. Some risks factors as adult age for *A. phagocytophilum* and breed (hunting breed for *A. phagocytophilum* and mixed breed for *E. canis*) were evidenced. This study also reports discordant results between different tests, showing that single diagnostic tests often fail to detect positive subjects in different stage of infection and confirming the importance of a diagnostic approach that combines molecular and serological tests. Considering a wider spread of the vectors due to climate change and an increasingly sharing of environment between companion animals and owners, constant surveillance of animal populations is necessary from a One Health perspective. The discovery of zoonotic strains in several animal cases suggests that companion animals can act as sentinels for human infections, and dogs with undiagnosed Anaplasmataceae infection may play an important epidemiological role by acting as source of infection for invertebrate hosts, with an increased risk of transmission to humans and posing a threat to public health.

### Electronic supplementary material

Below is the link to the electronic supplementary material.

Supplementary Material 1

Supplementary Material 2

Supplementary Material 3

Supplementary Material 4

Supplementary Material 5

## Data Availability

All data generated or analyzed during this study are included in this published article and its supplementary information files. The nucleotide sequences generated and analyzed during the current study are available in the International Nucleotide Sequence Database Collaboration repository (INSDC, http://www.insdc.org/) with the IDs: OR209777-OR209783.
