# Supplementary material for: Detection of Anaplasma spp. and Ehrlichia spp. in dogs from a veterinary teaching hospital in Italy: a retrospective study 2012–2020
Source: Vet Res Commun. 2024 Mar 27;48(3):1727–40. doi: 10.1007/s11259-024-10358-4 (PMC11147850; doi:10.1007/s11259-024-10358-4)
Supplement: Supplementary file 1 — Supplementary Material 1 [file 11259_2024_10358_MOESM1_ESM.pdf]

Veterinary Research Communication

Detection of *Anaplasma* spp. and *Ehrlichia* spp. in dogs from a veterinary teaching hospital in Italy: a retrospective study 2012-2020

Veronica Facile [a], Maria Chiara Sabetti [b], Andrea Balboni [a], Lorenza Urbani [a], Alessandro Tirolo [b], Martina Magliocca [a], Francesco Lunetta [a], Francesco Dondi [a*], Mara Battilani [a]

[a] Department of Veterinary Medical Sciences, *Alma Mater Studiorum*-University of Bologna, Via Tolara di Sopra 50, 40064 Ozzano dell'Emilia, Bologna, Italy

[b] Department of Veterinary Sciences, University of Parma, Strada del Taglio 10, 43126 Parma, Italy

* Corresponding author

Francesco Dondi

Department of Veterinary Medical Sciences, *Alma Mater Studiorum*-University of Bologna, Via Tolara di Sopra 50, 40064 Ozzano Emilia, Bologna, Italy

E-mail: f.dondi@unibo.it

Online Resource 1 Material and methods: clinicopathological investigations

Blood specimens were collected by standard venepuncture using blood vacuum collection systems (Vacutest Kima, Arzegrande, PD, Italy).

Samples were analysed within 1 hour after collection, and complete blood count (CBC), biochemistry profile, and urinalysis were carried out. CBC was carried out using an automated haematology analyser (ADVIA 2120, Siemens Healthcare Diagnostics, Erlangen, Germany). The haematology was completed with the microscopic blood smear examination using May-Grünwald Giemsa staining. Serum chemistry profile, including creatinine, urea, phosphate, total protein, albumin, globulin , alanine aminotransferase, aspartate aminotransferase (AST), alkaline phosphatase, gamma (g)-glutamyltransferase, total bilirubin, cholesterol, total calcium, sodium, potassium, chloride, magnesium and glucose, as reported previously by Troìa and colleagues (Troìa R, Balboni A, Zamagni S, Frigo S, Magna L, Perissinotto L, Battilani M, Dondi F (2018) Prospective evaluation of rapid point-of-care tests for the diagnosis of acute leptospirosis in dogs. Vet J 237: 37-42. https://doi.org/10.1016/j.tvjl.2018.05.010), was carried out using an automated analyser (AU480; Beckman Coulter-Olympus, Brea, CA, USA). Urinary protein and creatinine were also evaluated (AU480; Beckman Coulter-Olympus, Brea, CA, USA) and the urine protein to creatinine ratio (UPC) was calculated. Urine samples with a visible red colour and/or >250 red blood cells (RBCs) in a high-power field (hpf) were excluded from the UPC analysis.
