# Supplementary material for: Detection of Anaplasma spp. and Ehrlichia spp. in dogs from a veterinary teaching hospital in Italy: a retrospective study 2012–2020
Source: Vet Res Commun. 2024 Mar 27;48(3):1727–40. doi: 10.1007/s11259-024-10358-4 (PMC11147850; doi:10.1007/s11259-024-10358-4)
Supplement: Supplementary file 2 — Supplementary Material 2 [file 11259_2024_10358_MOESM2_ESM.pdf]

Veterinary Research Communication

Detection of *Anaplasma* spp. and *Ehrlichia* spp. in dogs from a veterinary teaching hospital in Italy: a retrospective study 2012-2020

Veronica Facile [a], Maria Chiara Sabetti [b], Andrea Balboni [a], Lorenza Urbani [a], Alessandro Tirolo [b], Martina Magliocca [a], Francesco Lunetta [a], Francesco Dondi [a*], Mara Battilani [a]

[a] Department of Veterinary Medical Sciences, *Alma Mater Studiorum*-University of Bologna, Via Tolara di Sopra 50, 40064 Ozzano dell'Emilia, Bologna, Italy

[b] Department of Veterinary Sciences, University of Parma, Strada del Taglio 10, 43126 Parma, Italy

* Corresponding author

Francesco Dondi

Department of Veterinary Medical Sciences, *Alma Mater Studiorum*-University of Bologna, Via Tolara di Sopra 50, 40064 Ozzano Emilia, Bologna, Italy

E-mail: f.dondi@unibo.it

Online Resource 2 Complete blood count results of dogs tested positive for *Anaplasma* spp. and *Ehrlichia* spp. infection

**Variables**	**RI**	**N**	**Positives**<br>**Median (range)**	**Positive to *A. ph***<br>**Median (range)**	**Positive to *A. pl***	**Positive to *E. ca***<br>**Median (range)**	**Positive to *A. ph* and *E. ca***<br>**Median (range)**	***P* value**
Hct (%)	39-58	63	37.8 (6-62.5)	44 (19-62.5)	28.4	39.6 (10-55.3)	27.8 (6-55.4)	0.1749
Hb (gr%)	14-19	63	12.3 (1.8-20.4)	14.5 (6.7-20.4)	9.2	13 (3.4-18.7)	9.1 (1.8-19)	0.2869
RBC (/mm3) x10^6	5.65-8.4	63	5.88 (075-43.8)	6.23 (27.8-77.7)	4.51	5.42 (2.83-8.06)	5.88 (0.75-43.8)	0.8593
MCV (fL)	63-77	63	67.4 (51.5-86)	69.2 (64-78.4)	63.1	66 (58.6-86)	66.2 (51.5-85.2)	0.0656
MCH (pg)	22-26	63	22.5 (15.4-28.2)	23.6 (20.7-24)	20.4	22.1 (18.1-28.2)	22.3 (15.4-26.2)	0.0842
MCHC (g%)	32-37	63	33.2 (29.8-36.6)	33.3 (31.3-34.9)	32.4	33.5 (30.1-36.6)	33.2 (29.8-36.5)	0.6888
RDW (%)	11.6-14.6	63	13.5 (10.9-34.6)	13.2 (11.8-18)	16.1	13.4 (11.2-34.6)	14.5 (10.9-29)	0.4905
Reticulocytes (/mm3)	0-120,000	63	31,500 (0.15-540,100)	28,800 (0.4-339,000)	237,100	41,000 (0.15-274,300)	30,100 (7500-540,100)	0.3179
PLT (/mm3) x10^4	15-50	63	16.3 (0.4-66.7)	26.35 (4.3-51)	28.2	16.4 (0.8-66.7)	12.9 (0.4-43)	0.1261
MPV (fL)	8.3-13.6	63	13.9 (7.9-23.8)	13.7 (8.5-18.8)	13.5	14.1 (7.9-23.3)	14.1 (9.2-23.8)	0.8278
Leukocytes (/mm3)	5000-14,000	63	8790 (260-76,350)	10,695 (1310-76,350)	12,590	7790 (1710-35,300)	8790 (260-25,770)	0.3211
Lymphocytes (/mm3)	900-4000	63	2060 (110-12,750)	1810 (300-12,750)	3650	2100 (420-5830)	2060 (110-5070)	0.5958
Monocytes (/mm3)	140-1000	63	530 (40-7290)	650 (90-7290)	800	460 (80-4142)	540 (40-1830)	0.3723
Neutrophils (/mm3)	3000-10,000	63	5150 (60-54,380)	6790 (320-54,380)	7350	4275 (410-12,967)	5340 (60-20,780)	0.2495
Eosinophils (/mm3)	10-1100	63	220 (10-4120)	285 (10-1080)	700	220 (10-4120)	110 (10-410)	0.1091
Basophils (/mm3)	0-80	63	40 (10-720)	50 (10-720)	40	50 (10-330)	30 (10-80)	0.3028

A. ph: *Anaplasma phagocytophilum*; *A. pl*: *Anaplasma platys; E. ca*: *Ehrlichia canis;* Hb: haemoglobin concentration; HCT: haematocrit value; MCH: mean corpuscular haemoglobin; MCHC: mean cell haemoglobin concentration; MCV: mean corpuscular volume; MPV: mean platelet volume; N: number of dogs for which the data were available; PLT: platelet count; RBC: red blood cell count; RDW: red cell distribution width; RI: reference interval
