# Supplementary material for: Detection of Anaplasma spp. and Ehrlichia spp. in dogs from a veterinary teaching hospital in Italy: a retrospective study 2012–2020
Source: Vet Res Commun. 2024 Mar 27;48(3):1727–40. doi: 10.1007/s11259-024-10358-4 (PMC11147850; doi:10.1007/s11259-024-10358-4)
Supplement: Supplementary file 3 — Supplementary Material 3 [file 11259_2024_10358_MOESM3_ESM.pdf]

Veterinary Research Communication

Detection of *Anaplasma* spp. and *Ehrlichia* spp. in dogs from a veterinary teaching hospital in Italy: a retrospective study 2012-2020

Veronica Facile [a], Maria Chiara Sabetti [b], Andrea Balboni [a], Lorenza Urbani [a], Alessandro Tirolo [b], Martina Magliocca [a], Francesco Lunetta [a], Francesco Dondi [a]*, Mara Battilani [a]

[a] Department of Veterinary Medical Sciences, *Alma Mater Studiorum*-University of Bologna, Via Tolara di Sopra 50, 40064 Ozzano dell'Emilia, Bologna, Italy

[b] Department of Veterinary Sciences, University of Parma, Strada del Taglio 10, 43126 Parma, Italy

* Corresponding author

Francesco Dondi

Department of Veterinary Medical Sciences, *Alma Mater Studiorum*-University of Bologna, Via Tolara di Sopra 50, 40064 Ozzano Emilia, Bologna, Italy

E-mail: f.dondi@unibo.it

Online Resource 3 Serum chemistry and urine protein to creatinine ratio (UPC) results of dogs tested positive for *Anaplasma* spp. and *Ehrlichia* spp. infection

**Variables**	**RI**	**N**	**Positives Median (range)**	**Positive to *A. ph* Median (range)**	**Positive to *A. pl***	**Positive to *E. ca* Median (range)**	**Positive to *A. ph* and *E. ca* Median (range)**	***P* value**
AST (U/L)	15-52	49	34 (1.12-2266)	32 (1.12-212)	52	34 (24-2266)	38 (23-1106)	0.4525
ALT(U/L)	15-65	61	41 (16-2846)	42.5 (18-703)	31	40 (16-1851)	45 (18-2846)	0.8621
ALP(U/L)	12-180	49	107 (17-1100)	179.5 (17-1100)	144	92 (19-1082)	51.5 (28-356)	0.8005
GGT (U/I)	0-5	37	2.3 (0.1-17.5)	3.1 (0.1-6.5)		2.25 (0.1-11)	1.25 (0.5-17.5)	0.2619
Glucose (mg/dL)	65-115	47	93 (32-120)	100 (32-113)	94	93 (66-120)	87.5 (80-119)	0.7682
Urea (mg/dL)	17-48	50	32 (14-393)	38.5 (14-393)	32	30.97 (14-122)	27 (15-247)	0.6772
Creatinine (mg/dL)	0.75-1.4	62	0.95 (0-11.68)	1.155 (0.51-11.68)	0.46	0.94 (0.25-2)	0.755 (0-5.35)	0.1166
Total bilirubin (mg/dL)	0.07-0.33	50	0.18 (0-17.33)	0.18 (0.11-17.33)		0.19 (0-13.68)	0.19 (0-0.29)	0.6912
Calcium (mg/dL)	9.7-11.0	37	10.7 (9.3-15)	10.7 (9.3-11.3)		10.65 (10-12.9)	10.65 (9.7-15)	0.7648
Phosphate (mg/dL)	2.65-5.40	41	4.26 (2.6-15.26)	4.26 (2.7-14.36)		4.05 (2.6-7.8)	5.45 (3.42-15.26)	0.0932
Sodium (mg/dL)	143-151	60	146 (137-160)	146.5 (142-160)	146	146 (137-149)	143 (137-150)	0.2145
Potassium (mEq/L)	3.8-5.0	62	4.4 (0.3-6.1)	4.45 (3-6.1)	4.4	4.4 (0.3-5.1)	4.65 (3.8-5)	0.8979
Chloride (mEq/L)	108-118	37	113.2 (11-122.7)	113 (11-119)		113.2 (105-117)	113.8 (107-122.7)	0.8958
Magnesium (mg/dL)	1.70-2.35	32	2 (1-3.59)	2.075 (1.84-2.96)		2 (1.6-3.59)	1.985 (1-2.63)	0.8480
Total protein (g/dL)	5.6-7.3	61	6.73 (3.57-14.39)	6.225 (4.47-7.68)	6.35	6.785 (3.57-14.39)	7.045 (5.81-10.74)	0.0614
Albumin (g/dL)	2.75-3.85	62	2.44 (1-3.74)	2.25 (1.29-3.71)	2.59	2.7 (1.19-3.61)	2.015 (1-3.74)	0.7128
Albumin to globulin ratio	0.75-1.35	62	0.595 (0-1.13)	0.65 (0.33-1.09)	0.6	0.77 (0.1-1)	0.445 (0-1.13)	0.3556
Total cholesterol (mg/dL)	123-345	36	211 (45-436)	221.5 (165-436)		205 (45-372)	200 (76-366)	0.6997
Urine protein to creatinine ratio	≤ 0.5	58	0.4 (0.1-21.1)	1.18 (0.1-21.1)	0.59	0.225 (0.1-15.58)	0.6 (0.11-14.9)	0.1038

A. ph: *Anaplasma phagocytophilum*; *A. pl*: *Anaplasma platys; E. ca*: *Ehrlichia canis;* RI: reference interval; ALP: alkaline phosphatase; ALT: alanine transaminase; AST: aspartate transaminase; GGT: γ-Glutamyl transferase; N: number of dogs for which the data was available
