# Supplementary material for: Detection of Anaplasma spp. and Ehrlichia spp. in dogs from a veterinary teaching hospital in Italy: a retrospective study 2012–2020
Source: Vet Res Commun. 2024 Mar 27;48(3):1727–40. doi: 10.1007/s11259-024-10358-4 (PMC11147850; doi:10.1007/s11259-024-10358-4)
Supplement: Supplementary file 4 — Supplementary Material 4 [file 11259_2024_10358_MOESM4_ESM.pdf]

Veterinary Research Communication

Detection of *Anaplasma* spp. and *Ehrlichia* spp. in dogs from a veterinary teaching hospital in Italy: a retrospective study 2012-2020

Veronica Facile [a], Maria Chiara Sabetti [b], Andrea Balboni [a], Lorenza Urbani [a], Alessandro Tirolo [b], Martina Magliocca [a], Francesco Lunetta [a], Francesco Dondi [a]*, Mara Battilani [a]

[a] Department of Veterinary Medical Sciences, *Alma Mater Studiorum*-University of Bologna, Via Tolara di Sopra 50, 40064 Ozzano dell'Emilia, Bologna, Italy

[b] Department of Veterinary Sciences, University of Parma, Strada del Taglio 10, 43126 Parma, Italy

* Corresponding author

Francesco Dondi

Department of Veterinary Medical Sciences, *Alma Mater Studiorum*-University of Bologna, Via Tolara di Sopra 50, 40064 Ozzano Emilia, Bologna, Italy

E-mail: f.dondi@unibo.it

Online Resource 4 Clinical signs of dogs tested positive for *Anaplasma* spp. and *Ehrlichia* spp. infection

**Clinical signs**	**N**	**Positives**	**Positive to *A. ph***	**Positive to *A. pl***	**Positive to *E. ca***	**Positive to *A. ph* and *E. ca***	***P* value**
Pale mucous membranes	66	15 (22.7%)	4	0	5	6	0.6364
Anorexia	66	33 (50%)	12	0	10	11	0.5920
Lymphadenomegaly	66	23 (34.8%)	6	0	10	7	0.5922
Cough	66	6 (9.1%)	3	0	1	2	0.5288
Depression	66	25 (37.9%)	9	0	9	7	0.9242
Joint pain/swelling	66	14 (21.2%)	6	0	3	5	0.4179
Neurological signs	66	2 (3.0%)	0	0	1	1	0.5895
Ocular signs	66	3 (4.5%)	0	0	2	1	0.3964
Nasal discharge	66	2 (3.0%)	0	0	1	1	0.5895
Melena	66	6 (9.1%)	1	0	3	2	0.6352
Dyspnoea	66	4 (6.1%)	2	0	1	1	0.7613
Presence of ticks	66	10 (15.2%)	3	0	5	2	0.5901
Fever*	66	28 (42.4%)	11	0	9	8	0.6692
PU/PD	66	13 (19.7%)	6	0	3	4	0.4526
Petechiae	66	3 (4.5%)	0	0	1	2	0.2972
Haematuria	66	3 (4.5%)	1	0	1	1	0.9947
Epistaxis	66	2 (3.0%)	1	0	0	1	0.5527
Vomit	66	10 (15.2%)	3	0	3	4	0.7648
Skin lesions	66	3 (4.5%)	0	0	1	2	0.2972
Diarrhoea	66	10 (15.2%)	3	0	4	3	0.9596
Weight loss	66	11 (16.7%)	6	0	3	2	0.2564
Abdominal pain	66	6 (9.1%)	2	0	2	2	0.9818
Congestive mucous membranes	66	2 (3.0%)	0	0	2	0	0.1645

A. ph: *Anaplasma phagocytophilum*; *A. pl*: *Anaplasma platys; E. ca*: *Ehrlichia canis;* N: number of dogs for which the data was available; PU/PD: polyuria/polydipsia

* Rectal temperature higher than 39.2° C (Miller JB (2014) Hyperthermia and fever. In: Silverstein DC, Hopper K (eds) Small Animal Critical Care Medicine, 2nd edn. Elsevier, St. Louis, pp. 55-59).
