# Supplementary material for: Detection of Anaplasma spp. and Ehrlichia spp. in dogs from a veterinary teaching hospital in Italy: a retrospective study 2012–2020
Source: Vet Res Commun. 2024 Mar 27;48(3):1727–40. doi: 10.1007/s11259-024-10358-4 (PMC11147850; doi:10.1007/s11259-024-10358-4)
Supplement: Supplementary file 5 — Supplementary Material 5 [file 11259_2024_10358_MOESM5_ESM.pdf]

Veterinary Research Communication

Detection of *Anaplasma* spp. and *Ehrlichia* spp. in dogs from a veterinary teaching hospital in Italy: a retrospective study 2012-2020

Veronica Facile [a], Maria Chiara Sabetti [b], Andrea Balboni [a], Lorenza Urbani [a], Alessandro Tirolo [b], Martina Magliocca [a], Francesco Lunetta [a], Francesco Dondi [a]*, Mara Battilani [a]

[a] Department of Veterinary Medical Sciences, *Alma Mater Studiorum*-University of Bologna, Via Tolara di Sopra 50, 40064 Ozzano dell'Emilia, Bologna, Italy

[b] Department of Veterinary Sciences, University of Parma, Strada del Taglio 10, 43126 Parma, Italy

* Corresponding author

Francesco Dondi

Department of Veterinary Medical Sciences, *Alma Mater Studiorum*-University of Bologna, Via Tolara di Sopra 50, 40064 Ozzano Emilia, Bologna, Italy

E-mail: f.dondi@unibo.it

Online Resource 5 Results obtained by the three different assays in dogs tested positive

Dogs	RIT	IFAT	PCR
01	NT	+	NT
02	NT	+	NT
03	NT	+	NT
04	NT	+	NT
05	NT	+	NT
06	NT	+	NT
07	NT	+	NT
08	NT	+	NT
09	NT	+	NT
10	NT	+	NT
11	NT	+	NT
12	NT	+	NT
13	NT	+	NT
14	NT	+	NT
15	NT	+	NT
16	NT	+	NT
17	NT	+	NT
18	NT	+	NT
19	NT	NT	+
20	NT	NT	+
21	NT	+	NT
22	NT	+	NT
23	NT	+	NT
24	NT	+	NT
25	NT	+	NT
26	NT	+	NT
27	NT	+	NT
28	NT	+	NT
29	NT	+	NT
30	NT	NT	+
31	NT	+	NT
32	NT	+	NT
33	NT	+	NT
34	NT	+	NT
35	NT	+	NT

36	NT	+	NT
37	NT	+	NT
38	NT	+	NT
39	NT	+	NT
40	+	NT	NT
41	+	NT	NT
42	+	NT	NT
43	+	NT	NT
44	+	-	NT
45	+	-	NT
46	-	NT	+
47	-	+	NT
48	+	+	NT
49	+	+	NT
50	+	+	NT
51	+	+	NT
52	-	+	NT
53	+	+	NT
54	+	+	NT
55	+	+	NT
56	+	+	NT
57	-	+	NT
58	+	+	NT
59	+	+	NT
60	+	NT	+
61	+	NT	+
62	+	NT	-
63	NT	+	+
64	NT	+	-
65	NT	+	+
66	NT	+	+
67	NT	+	-
68	NT	+	-
69	NT	+	-
70	-	NT	+
71	NT	+	-
72	NT	+	-

73	NT	+	-
74	+	+	-
75	+	+	-
76	+	+	-
77	+	+	-
78	+	+	-
79	+	+	+
80	+	+	-
81	+	+	+
82	-	-	+
83	+	+	-
84	+	+	-
85	+	+	-
86	+	+	-
87	+	+	-
88	+	+	+
89	+	+	-
90	-	+	-
91	+	+	+
92	+	+	-
93	+	+	-
94	+	+	-

IFAT: indirect fluorescent antibody test; NT: not tested; PCR: polymerase chain reaction, RIT: rapid immunoenzymatic test; +: positive result; -: negative result
